# An “exceptional” magnetic sensor

**DOI:** 10.1038/s41377-025-02005-7

**Published:** 2025-10-11

**Authors:** Zhenhuan Yi, Girish S. Agarwal, Marlan O. Scully

**Affiliations:** Institute for Quantum Science and Engineering, Texas A&M University, College Station, TX 77843 USA

**Keywords:** Magneto-optics, Optical sensors, Quantum optics, Imaging and sensing

## Abstract

Building a sensitive magnetic field sensor is non-trivial; building a more sensitive one by adding extra loss to the sensor is counterintuitive, but with innovative ideas from non-Hermitian physics like an exceptional point, a new magnetic field sensor first of its kind paves the way for broader applications of similar techniques.

Magnetic sensors are quite commonly used in our everyday life. Most widely used are, for example, those in cell phones, providing compass and orientation for navigation. Hundreds of millions of hard disk drives are sold every year, and key components of these devices are delicate magnetic sensors, which read and write data in the magnetic-based disc media. Cars and many vehicles use magnetic sensors to measure rotation of wheels to determine milage, to detect proximity of doors to control lights, etc. Of course, a lot of scientific studies use highly sensitive magnetic sensors to detect and study objects from small (e.g., atoms) to big (e.g., the earth).

In recent years, exceptional point (EP) physics has attracted a lot of attention in the scientific community for its potential to enhance sensitivity of a measuring system^1^, this was realized in, for example, an optical cavity^2^ and recently, a mechanical system^3^. While a Hermitian system always has real eigenvalues, a non-Hermitian system does not, and this opens doors for engineered systems to show special properties that can be used for various purposes.

Recently, Y. Ruan et al. demonstrated an innovative method to construct a tunable magnetic sensor, which is based on exceptional point property of a non-Hermitian system^4^.

The work published in Nature Photonics uses magneto-optical (MO) effect, which most people get to know through the Faraday effect or Faraday rotation: linearly polarized light propagating through MO material accumulates rotation of polarization proportional to the magnetic field strength applied to the material along the direction of propagation of the light. The effect is due to refractive index (thus propagation speed) differences introduced by the magnetic field between two orthogonal circular polarizations of light.

The authors bring in novelty by putting the MO material into an optical cavity to form a Hermitian system. Intuitively, the frequencies of the cavity modes are reverse proportional to the optical path (the refractive index times the length of a material), therefore they can be tuned by the refractive indices of the MO material on two orthogonal polarizations as the magnetic field is applied. As shown in Fig. 1a, the mode splitting increases proportionally with the magnetic field in this case. The MO effect also provides coupling between the two linear polarizations of light. To further construct a non-Hermitian system, a tunable liquid crystal absorber, which introduces polarization-dependent losses, was inserted into the cavity (see Fig. 1b). With careful derivations and proper assumptions, the resulting interaction Hamiltonian $$H=h\left(\begin{array}{cc}{\nu }_{H}-i{\kappa }_{H} & {igB}\\ -{igB} & {\nu }_{V}-i{\kappa }_{V}\end{array}\right)$$ has eigenfrequencies exhibiting an EP when ${gB}=\left({\kappa }_{H}-{\kappa }_{V}\right)/2$ and ${\nu }_{H}={\nu }_{V}$, where $\nu$’s are frequencies, $g$ is a coupling constant, $B$ is magnetic field strength, $\kappa$’s are polarization-dependent losses introduced by the liquid crystal absorber and $i=\sqrt{-1}$.

**Fig. 1:**
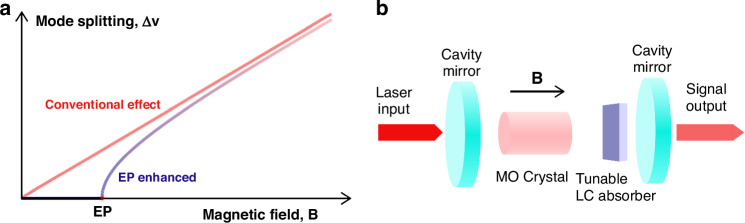
Magnetic field sensing via exceptional point enhanced magneto-optical effect. **a** The conventional magneto-optical (MO) effect and the exceptional point (EP) enhanced MO effect. The mode splitting is suppressed when the magnetic field strength is below EP and accelerated after passing EP. **b** Schematics of the experimental system. Linearly polarized laser light is injected into the cavity; the MO crystal couples two linear polarization components of light while the tunable liquid crystal (LC) absorber induces polarization-dependent losses. The output modes are thus elliptically polarized and require mode analysis

In this case, as the $B$ field increases from zero, the frequency splitting of the two eigenmodes is zero when ${gB} < \left({\kappa }_{H}-{\kappa }_{V}\right)/2$, and jumps to a square-root increase at the EP, as sketched in Fig. 1a. This square-root dependence facilitates a larger frequency splitting in response to $B$ field change near the EP. On the other hand, since the LC absorber provides a tunable loss on V-polarization, the EP position is tunable and makes the system a tunable magnetic sensor once calibrated. The error sensitivity can improve several times from the value of the conventional sensor. While the experiment is in the domain of classical electrodynamics, one can improve further the sensitivity by using quantum probes.

One important finding of this work is that with pure passive losses, it shows a clearly enhanced sensitivity near EP when other gain-loss type EP systems so far do not. The clear results may partially be attributed to the way the two modes are measured: due to the coupling and losses, the two modes become elliptically polarized, therefore hard to separate via polarization; but the team managed to estimate the two modes via double Lorentzian line shape analysis and fittings and demonstrates enhancement under different tunable EP conditions.

Many systems can be modelled in terms of a non-Hermitian Hamiltonian describing two coupled oscillators: $$\left(\begin{array}{cc}{\omega }_{1}-i{\gamma }_{1} & {g}_{1}\\ {g}_{2} & {\omega }_{2}-i{\gamma }_{2}\end{array}\right)$$, where the $\omega$’s are energy of the two oscillators, $\gamma$’s are decay rates, $g$’s are coupling strength from one to the other oscillator^5,6^. Generally, $\gamma$’s and $g$’s are non-equal. While this work, inspired by studies on PT symmetric systems, engineered different $\gamma$’s to use the EP physics to enhance sensitivity, anti-PT symmetry systems as well offer EP’s that can be utilized for sensitivity enhancement^6^. Moreover, different $g$’s also offer rich physics such as time crystal formation^7^, and quantum amplification by superradiant emission of radiation^8^.

The techniques used in the work from K. Xia’s group can be adapted to other optomagnetic systems, e.g., optically pumped atomic systems and, as the authors pointed out, fiber-based magnetic field sensors, to enhance the sensitivity of those systems. The latter indeed has huge potential in the emerging field of optical fiber-based wearable devices and fabrics^9^, high sensitivity wearable devices can potentially impact healthcare, e.g., brain function studies and diagnostics.
